# Aqueous Extract of Black Maca (*Lepidium meyenii*) on Memory Impairment Induced by Ovariectomy in Mice

**DOI:** 10.1093/ecam/nen063

**Published:** 2011-02-14

**Authors:** Julio Rubio, Wang Qiong, Xinmin Liu, Zhen Jiang, Haixia Dang, Shi-Lin Chen, Gustavo F. Gonzales

**Affiliations:** ^1^Research Center for Pharmacology and Toxicology, Institute of Medicinal Plant Development, Chinese Academy of Medical Sciences and Peking Union Medical College, Beijing 100193, China; ^2^Department of Biological and Physiological Sciences, Faculty of Sciences and Philosophy and Instituto de Investigaciones de la Altura, Universidad Peruana Cayetano Heredia, PO Box 1843, Lima, Peru

## Abstract

The present study aims to test two different doses of aqueous extract of black maca on learning and memory in ovariectomized (OVX) mice and their relation with malonalehyde (MDA), acetylcholinesterase (Ache) and monoamine oxidase (MAO) brain levels. Female mice were divided into five groups: (i) naive (control), (ii) sham, (iii) OVX mice and OVX mice treated with (iv) 0.50 g kg^−1^ and (v) 2.00 g kg^−1^ black maca. Mice were orally treated with distilled water or black maca during 35 days starting 7 days after surgery. Memory and learning were assessed using the water Morris maze (from day 23–27) and the step-down avoidance test (days 34 and 35). At the end of each treatment, mice were sacrificed by decapitation and brains were dissected out for MDA, Ache and MAO determinations. Black maca (0.5 and 2.0 g/kg) increased step-down latency when compared to OVX control mice. Black maca decreased MDA and Ache levels in OVX mice; whereas, no differences were observed in MAO levels. Finally, black maca improved experimental memory impairment induced by ovariectomy, due in part, by its antioxidant and Ache inhibitory activities.

## 1. Introduction

*Lepidium meyenii* Walp., known as maca, grows over 4000 m altitude in the central Peruvian Andes. Previous studies were focused to demonstrate the traditional fertility-enhancing properties of the hypocotyls of maca [1–3]. In addition, maca has been demonstrated to have antioxidant properties *in vitro* and *in vivo* [4, 5].

Epidemiological studies found a significant correlation between dietary intake of vegetables and improvement in cognitive function in elderly people [6]. For instance, aging women consuming cruciferous vegetables (e.g., broccoli and cauliflower) showed less cognitive decline than those not consuming them [7]. Members of the genus *Lepidium*, including maca, belong to the cruciferous (Brassicaceae) family and it is possible that this plant may have effects on cognitive functions. In fact, many reports have claimed other non-traditional properties of maca including its capacity to reduce menstrual and menopausal symptoms in women, promote mental clarity, restore hormonal balance in women and improve memory [8–10]. Previously, when different varieties of maca (red, yellow and black) were compared for its capacity to induce memory improvement, black maca showed the greatest effect [11]. In addition, it was demonstrated that black maca improve memory impairment induced by scopolamine, a muscarinic cholinergic receptor antagonist [12].

Ovariectomy is a well-known animal model to induce memory impairment in rodents [13]. It was previously demonstrated that ovariectomized (OVX) rodents showed a lower performance in spatial tests such as water Morris maze and eight-arm radial maze [14] and reduced latent time in the step-down avoidance test [15]. Moreover, other studies showed that the deleterious effect of ovariectomy on memory could be related to its capacity to induce oxidative stress [16], cholinergic and monoaminergic dysfunction [17, 18] in brain.

Considering that: (i) maca is used for its capacity to improve memory, (ii) maca has antioxidant activity, (iii) black maca shows the greatest effect on cognitive function, (iv) black maca showed acetylcholinesterase (Ache) inhibitory activity and no effect on monoamine oxidase (MAO) levels in male mice and (v) ovariectomy is an female animal model that induce memory impairment related to an increased oxidative stress and cholinergic and MAO dysfunction, the present study aims to evaluate the effect of black maca in ovariectomy-induced memory impairment in female mice and its effect on malonalehyde (MDA), Ache and MAO brain levels.

## 2. Subjects and Methods

### 2.1. Animals

Female mice of 3-months old from the Kunming strain (28.29 ± 0.28 g) were used for the study. Mice were maintained at ambient temperature (23.0 ± 2.0°C) with a 12 : 12 h light/dark cycle in the animal house of the Institute of Medicinal Plant Development (IMPLAD). Mice were provided with laboratory chow and tap water *ad libitum*. All animal experiments were conducted in compliance with “Guide of the care and use of laboratory animals" [19].

### 2.2. Ovariectomy

Naive mice were anesthetized with 50 mg kg^−1^ of pentobarbital (ip). Bilateral ovariectomies were performed using a dorsolateral approach. Ovaries and surrounding fat tissue were removed and the incision was closed by suturing the muscles and skin. Similar surgical procedures were carried out for the sham operated animals except that the ovaries were not removed. Experiments were performed one week (7 days) after they were ovariectomized.

### 2.3. Preparation of Aqueous Extract of Black Maca

The dried hypocotyls of black maca were obtained from Carhuamayo, Junin at 4000 m altitude. Irma Fernandez, a botanist of the Department of Pharmaceutical Sciences, Universidad Peruana Cayetano Heredia, authenticated the identity of the plant (voucher number IFV1885). The aqueous extract was prepared according to the traditional method. In brief, the pulverized dried hypocotyls were placed in a container with water and boiled. The preparation will be left standing to cool, filtered, freezed (−70°C) and lyophilized (Lyophilizer freeze Mobile12). One gram of dried black maca hypocotyls produced 0.46 g of lyophilized black maca.

### 2.4. Treatments

In all experiments, a feeding needle N°18 (Fisher Scientific, Pittsburgh, PA, USA) for oral administration was used to administer aqueous (0.5 and 2.0 g kg^−1^) extract of black maca or vehicle (distilled water) for 35 days between 9:00 h and 12:00 h. Animals were divided in the following groups (*n* = 10): naive, sham, OVX control groups and two groups of OVX mice treated with two doses aqueous extract of black maca (0.5 and 2.0 g kg^−1^). No differences between groups regarding body weight were observed at the beginning of the experiment (data not shown).

### 2.5. Water Morris Maze

This task was adapted for mice from the paradigm originally described by Morris [20]. The water maze was a circular pool (65 cm diameter, 25 cm deep), filled with water (26 ± 1°C) and made opaque with black ink, to the depth of 20 cm. The pool was divided into four quadrants. An escape platform was placed in the middle of one quadrant, 1.0 cm below the water surface, equidistant from the sidewall and middle of the pool. The platform providing the only escape from the water was located in the same quadrant on every trial. Three different starting points for mice were placed around the perimeter of the pool. On each of the four training days, all three start points were used once each in a pseudo-random sequence, so the starting point was different in every session. All assessments were performed using a CRE camera that was suspended over the center of the pool. The swimming activity of each mice was recorded using an automated tracking system (China's Cosmonaut Training Center and Institute of Medicinal Plant Development) [21] coupled to a personal computer. The water maze was always located in a large room and the experimenter was always sat at the same position. All experiments were carried out between 10:00 h and 16:00 h.

#### 2.5.1. Escape Acquisition

A trial began by placing the animal in the water facing the wall of the pool at one of the starting points. If the animal failed to escape on the platform within 120 s, it was gently placed there by the experimenter and allowed to stay for 15 s. The inter-trial interval was 5–10 min. Three escape trails were given to all mice per day for four consecutive days (days 23–26 of each treatment). The escape latency(s), swim distance and average speed (cm s^−1^) to reach the platform were recorded during these trials.

#### 2.5.2. Spatial Memory Test

Twenty four (day 27) hours after the last training trial in the escape acquisition test, mice were submitted to the probe trial in which the platform was removed. In the 60-s probe trial, the time in the target quadrant (s; the quadrant in which the platform was located in the training sessions) and the number of target crossings (number of crossings over the former location of the platform) were obtained as a measure for spatial memory.

### 2.6. Step-Down Avoidance Test

The apparatus was a plastic box (27 × 15 × 12 cm^3^) whose floor was made of parallel bronze bars. The left end of the grid was occupied by a 4 cm diameter, 5 cm high wooden platform. The behaviour of mice was recorded in a personal computer using an automated tracking system (China's Cosmonaut Training Center and Institute of Medicinal Plant Development; [22]) coupled to an infrared sensor located in the apparatus. The experiments were carried out from 10:00 to 14:00 h.

The step-down avoidance test was performed 6 days after the Morris water maze as previously described [23]. Before the beginning of the training session, mice were placed on the apparatus to adapt for 3 min. In the training session (day 34), mice were put on the grid floor and then a continuous electric shock (0.4 mA) was delivered to the grid floor by an isolated stimulator. When the electric shock was delivered, mice escape from the grid floor back up onto the platform. The duration of training test was for 5 min and the shock was maintained for this period. Twenty-four hours after training (day 35), mice were placed on the platform for the retention test. The electric shocks were still delivered for 5 min. Step-down latency and the number of error were recorded with improved retention reflected by increased latency and reduction in errors.

### 2.7. Biochemical Determination of MDA, Ache and MAO Levels

At the end of the experiments, mice were sacrificed by decapitation and the brains were dissected out. The brains were homogenized in 10.0 ml of saline (NaCl 0.9%), centrifuged at 3500 g for 15 min and the supernatant obtained for biochemical determinations (MDA, Ache and MAO levels). The determination of MDA, Ache and MAO levels were performed using commercial kits (Nanjing Jiancheng Bioengineering Institute, P.R. China). All samples were run in a same assay to avoid between-assay variation. Protein concentrations were determined by the Lowry method [24] using bovine serum albumin as a standard.

### 2.8. Statistical Analysis

Data were analyzed using the statistical package STATA 8.0 for personal computer (Stata Corporation, Texas, USA). Data are presented as mean ± SEM. Homogeneity of variances was assessed using a Bartlett test. Variables with homogeneous variances (body weights and those corresponded to water Morris maze) were analyzed by analysis of variance (ANOVA). If the *P*-value in the ANOVA test was significant, *post hoc* Scheffé test was run when indicated. When variances were not homogeneous (latency and number of errors in the step-down test and MDA, Ache and MAO brain levels), the Kruskal-Wallis test was used to assess differences between groups. If the result was statistically significant, differences between pair of medians were assessed using the Mann-Whitney *U*-test. A value of *P* < .05 was considered to be statistically significant.

## 3. Results

### 3.1. Body Weight

No differences among groups were observed in body weight at the beginning of the experiments (*F*_4,44_ = 1.80, *P* = NS). At the end of the study, differences in body weight gain were observed between groups (*F*_4,44_ = 4.68, *P* < .05). OVX mice showed a higher body weight increase than mice in naive (14.65%; *P* < .05) and sham (16.70%; *P* < .05) groups. Black maca did not alter the effect of ovariectomy on body weight (24.74 and 22.29% for 0.5 and 2.0 g kg^−1^ black maca, respectively; *F*_4,44_ = 4.68, *P* > .05). In fact, the increase in body weight in OVX mice treated with black maca was higher than naive and sham groups (*P* < .05).

### 3.2. Effect of Black Maca on Escape Acquisition and Spatial Memory in OVX Mice

**Figure 1:**
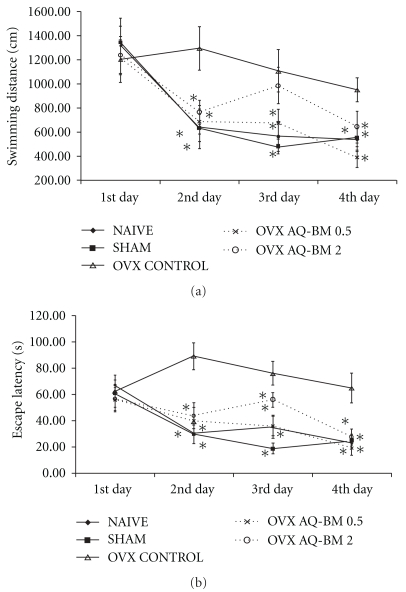
Black maca on (a) swimming distance (centimeters) and (b) escape latency (s) in OVX mice during the escape acquisition test of the water Morris maze. Data are presented as mean ± SEM. *P < .05 versus OVX control.

During the acquisition test, no differences between groups were observed during the first day in swimming distance (*F*_4,44_ = 0.13, *P* = NS). When OVX control group was compared against naive and sham groups, OVX mice showed longer swimming distance than naive and sham groups during days 2 (*F*_2,26_ = 5.27, *P* < .05), 3 (*F*_2,26_ = 6.81, *P* < .05) and 4 (*F*_2,26_= 5.29, *P* < .05). No differences between mice in naive and sham groups were observed (*P* > .05). From day 2 to 4, mice treated with 0.5 and 2.0 g kg^−1^ of black maca showed a reduction in swimming distance than OVX mice in control group reaching similar values to those of naive and sham groups (*F*_4,44_ = 3.73, *P* < .05; *F*_4,44_ = 4.29, *P* < .05; *F*_4,44_ = 3.78, *P* < .05 for days 2, 3 and 4, resp.) (Figure 1(a)). 

All groups showed no significant changes in escape latency during the first day (*F*_4,44_ = 0.12, *P* > .05). Ovariectomy significantly increased the time to reach the fixed platform with respect to naive and sham mice in days 2 (*F*_2,26_ = 15.22, *P* < .05), 3 (*F*_2,26_ = 14.25, *P* < .05) and 4 (*F*_2,26_ = 9.85, *P* < .05). Black maca reduced the escape latency when compare to the OVX control group during day 2 (*F*_4,44_ = 6.53, *P* < .05), 3 (*F*_4,44_ = 8.98, *P* < .05) and 4 (*F*_4,44_ = 7.07, *P* < .05) (Figure 1(b)).

Average speed was not affected by any treatment (*F*_4,44_ = 1.87, *P* = NS).

**Figure 2:**
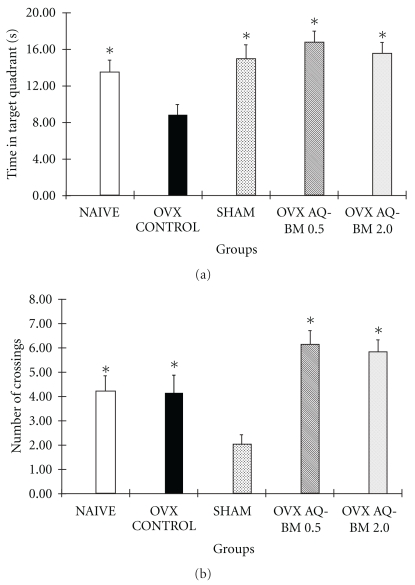
Black#maca on (a) the time in the target quadrant (s) and (b) number of crossings in OVX mice in the Morris water maze during the spatial memory test. Data are presented as mean ± SEM. *P < .05 versus OVX control.

OVX mice showed lower time in the target quadrant (*F*_2,26_ = 5.69, *P* < .05) and lower number of crossings (*F*_2,26_ = 4.09, *P* < .05) than mice in naive and sham groups. Both doses of black maca prevented the decrease in the time spent in the target quadrant (*F*_4,44_ = 5.58, *P* < .05) and the number of crossings of the previous location of the platform (*F*_4,44_ = 7.47, *P* < .05) (Figures 2(a) and 2(b)).

### 3.3. Effect of Black Maca on Step-Down Avoidance Test in OVX Mice

Non-parametric analyses showed that OVX mice presented lower step-down latency than mice in naive and sham groups (*P* < .05). OVX mice treated with both doses of black maca showed higher values of step-down latency than OVX mice treated with vehicle (*P* < .05) (Table 1).

### 3.4. MDA, Ache and MAO Brain Levels

MDA and Ache brain levels were increased in OVX mice when compare with naive and sham groups (*P* < .05). No differences were observed between naive and sham groups (*P* > .05). OVX mice receiving two different doses of black maca showed lower MDA and Ache brain levels than OVX mice (*P* < .05). MDA and Ache levels in black maca groups were similar than values in control groups (naïve and sham). OVX mice treated with 0.5 g kg^−1^ of black maca showed higher Ache values when compare with naive group (*P* < .05). No differences in MDA and Ache levels were observed in OVX mice treated with black maca (*P* > .05) (Table 2). In addition, no differences in MAO levels were observed between groups (*P* > .05).

MDA, Ache and MAO values for each group were log-transformed for correlation analyses to determine the relation between these variables and those from the memory tests. Negative correlations were observed between MDA and Ache values and total time in the target quadrant (*r* = −0.48, *P* = .002; *r* = −0.36, *P* = .021, resp.) and number of crossings (*r* = −0.47, *P* = .003; *r* = −0.43, *P* = .005, resp.) in the spatial probe of the water Morris maze. No correlations were observed between MDA and Ache brain levels and latency (*r* = 0.04, *P* = .83; *r* = −0.03, *P* = .872, resp.) and number of errors (*r* = 0.29, *P* = .08; *r* = 0.28, *P* = .083, resp.) in the step-down test.

## 4. Discussion

Maca is naturally present in different varieties which are characterized by their external color and different biological effects were described for yellow, red and black maca [25]. In fact, the effect of black maca on memory was previously elucidated using OVX mice [11].

Ovariectomy is characterized by progressive memory deficits, central cholinergic nerve system degeneration, excessive oxidative stress and differentiation/apoptosis imbalance [26]. This model has been a widely used *in vivo* model to mimic post-menopausal pathophysiological changes in women related to learning and memory [14], brain oxidative stress [13] and cholinergic and monoaminergic function [18, 26]. In the present study, ovariectomy resulted in impairment on memory function as observed in the water Morris maze and step-down avoidance tests. These results are in accordance with those described in previous studies [15]. Although hormone deprivation due to ovariectomy causes spatial memory deficits, it is important to notice that estrous cycle may influence the performance in memory tests [27]. We do not discard the possibility that gonadal steroids or other substances produced by ovaries may have some influence on our results, the lack of differences between gonadally intact and OVX groups during the first day of training in the water Morris maze and step-down avoidance test suggests that gonadal estrogens are not biasing our results. In addition, a possible estrogenic activity of black maca on memory should be discarded due to the fact that it was previously demonstrated that black maca did not have estrogenic activity [25].

In the water Morris maze, black maca was able to alleviate the effect of ovariectomy. It is important to notice that water Morris maze investigated spatial learning and memory [28]. From this, it is reasonable to suggest that black maca may improve spatial learning and memory deficits induced by ovariectomy. During the step-down avoidance test, black maca in OVX mice increased latency time and reduced the number of errors. Although black maca did not fully reverse the effect of ovariectomy during the test, black maca treatment partially attenuates its effect by giving to OVX mice similar behaviors to naive and sham mice. Step-down latency is taken as a measurement of retention [9] and for this reason it is possible to suggest that black maca diminished the effect of ovariectomy related to retention.

Antioxidants play an important role in preventing or alleviating chronic diseases by reducing the oxidative damage to cellular components caused by reactive oxygen species [29]. Naturally occurring polyphenolic constituents are potential antioxidants [30]. For this reason, researchers focused in the finding and screening of natural products from different plant species to identify new antioxidants in order to avoid synthetic antioxidant products and their secondary toxic effects [31, 32]. In addition, the brain is susceptible to be peroxidated by free radicals [33]. Lipid peroxidation (LPO) readily decomposes to liberate carbonyl fragments, the most prominent being MDA, which are highly reactive and responsible for cytotoxic effects and neuronal death [33]. From this, MDA levels can be taken as an indicator for the state of LPO. The reported abnormal alteration in MDA levels and its relation to memory impairment have been showed in previous studies [34]. Our results confirmed the capacity of black maca to reduce oxidative stress. So, black maca may improve learning and memory in OVX mice by its capacity to reduce oxidative stress.

Cholinergic dysfunction is the primary effect observed in progressive loss of memory and dementia in the elderly (i.e., Alzheimer disease) [35]. For instance, Ache inhibitors are the first group of compounds for Alzheimer disease treatment [36]; however, long-term treatment can cause adverse side effects (mainly by the activation of peripheral cholinergic systems; [37]). The latter open the opportunity to search new long-acting Ache inhibitors with minimal clinical side effects. In fact, previous studies observed that green tea polyphenols exhibited an inhibitory effect on Ache activity and significantly reversed scopolamine-induced retention deficits, both step-through passive avoidance and spontaneous alternation behavior tasks [38]. Outcomes from the present study showed that black maca present Ache inhibitory activity in OVX mice. This finding is supported by the fact that black maca reduced Ache activity in mice treated with scopolamine [12].

As observed previously [12], results from the present study showed that black maca extracts did not alter MAO activity. From this, it is suggested that the neuroprotective effect of black maca do not result from direct MAO inhibition as observed with other products [39, 40]. This finding is important since it has been suggested the possibility that (1*R*,3*S*)-1-methyltetrahydro-*β*-carboline-3-carboxylic acid, a molecule which is reported to be present in maca, may affect the central nervous system negatively [41]. Tetrahydro-*β*-carbolines arises from a Pietet-Splenger condensation between l-tryptophan and aldehydes and this reaction is temperature- and pH-dependent [42]. The latter makes it possible to suggest that the traditional preparation of maca may be not related to the occurrence of tetrahydro-*β*-carbolines in the aqueous extract. Data from this study and that from others [11, 12] suggest that black maca may be neuro-protective.

The neuro-protective effects of plants containing polyphenolic compounds such as quercetin [43] and anthocyanins [44] have been previously reported. Moreover, it has been demonstrated that polyphenolic compounds have Ache inhibitory activity and antioxidant effect on brain [38, 45, 46]. It was previously demonstrated that polyphenolic compounds are able to cross the blood brain barrier and localize in various brain regions (including cerebellum, cortex, hippocampus or striatum) being this localization in brain significantly correlated with good performances in the water Morris maze and passive avoidance tasks [47]. Previous studies demonstrated that maca hypocotyls contain polyphenolic compounds such as quercetin [5] and anthocyanins [48]. The compounds in black maca related to its neuroprotective effect have not been elucidated yet, but it is suggested that the effect of black maca on memory and learning may be due to its content of polyphenolic compounds such as quercetin and anthocyanins.

In the present study, 7 days after ovariectomy no differences in body weight were observed between groups as previously reported [49]. An increase in body weight in OVX mice was observed at the end of the experiments. These results are in accordance with other authors that found that body weight in OVX animals was increased after 4 and/or 6 weeks after surgery due to hyperphagia [50]. Similar to our results, Zhang et al. [51] found that maca did not alter the body weight increase due to ovariectomy. Other authors found that estradiol and soy phytoestrogens significantly decreased body weight and adipose tissue deposition by increasing metabolism [52]. The latter is in accordance with the previous finding that maca had neither estrogenic nor androgenic activities *in vivo* [25, 52].

Finally, these results demonstrated that black maca can enhance learning and memory in OVX mice and this effect might be related, at least in part, to its ability to reduce LPO and Ache in OVX mice.

## Funding

National Nature Science Foundation of China, NSFC in 2004 (30472016/C03020701); International Technologic Collaboration Project, grant no. 2006DFA21740; Peruvian National Council of Sciences, Technology and Innovation (CONCYTEC) through the grant PROCOM 2005.

## Figures and Tables

**Table 1:** Latency (s) in OVX mice treated with black maca during the step-down avoidance test.

Treatment	Dose	Number of errors	Step-down latency(s)
Native control	—	2.44 ± 0.73* [9]	93.33 ± 26.30* [9]
Sham control	—	2.11 ± 0.95* [10]	124.85 ± 34.81* [10]
OVX control	—	9.44 ± 2.15 [9]	29.69 ± 5.65 [9]
Aqueous extract of Black Maca	0.50 g/kg	3.67 ± 0.87* [10]	106.07 ± 23.21* [10]
	2.00 g/kg	4.37 ± 0.68* [10]	73.43 ± 13.89* [10]

Number of mice per group is in parenthesis.

*P < .05 versus OVX control.

**Table 2:** The effect of black maca on MDA, Ache and MAO levels on OVX mice brain.

Groups	Done	MDA (nmd/mgport)	Ache activity (U/mgprot)	MAO activity (U/ h/mgprot)
Naive control	–—	5.77 ± 1.80* [8]	5.32 ± 1.94* [7]	17.74 ± 4.71 [9]
Shan control	—	8.53 ± 0.87* [7]	8.37 ± 0.44* [9]	18.74 ± 5.18 [9]
OVX control	—	13.03 ± 1.10 [9]	14.34 ± 1.66 [8]	18.02 ± 4.28 [9]
OVX black maca	0.5 g/kg	5.64 ± 0.67* [7]	9.89 ± 0.54^∗(a)^ [9]	18.97 ± 4.67 [9]
	2.0 g/kg	5.32 ± 0.58* [8]	8.05 ± 0.59* [9]	17.98 ± 4.52 [9]

Number of mice per group is in parenthesis.

*P < .05 and ^(a)^ *P* < .05 versus OVX and naive groups, respectively.
